# Meta-analysis of changes in thiol–disulfide homeostasis during preeclampsia

**DOI:** 10.17305/bb.2023.9430

**Published:** 2024-02-01

**Authors:** Dan Wang, Guihong Yang, Xinxin You, Zijuan Zhang

**Affiliations:** 1 Department of Obstetrics, Taizhou Hospital of Zhejiang Province affiliated to Wenzhou Medical University, Taizhou, China; 2 Department of Ultrasonography Lab, Taizhou Hospital of Zhejiang Province affiliated to Wenzhou Medical University, Taizhou, China

**Keywords:** Thiol, disulfide, preeclampsia (PE), oxidative stress, meta-analysis

## Abstract

The present study systematically assessed alterations in thiol–disulfide homeostasis among women with preeclampsia (PE) through meta-analysis. This was conducted as such changes are believed to be associated with the oxidative stress underlying this condition. A comprehensive search of Medline, Web of Science, and Embase databases was conducted from their inception until 22 March 2023, to identify studies comparing levels of native thiol, total thiol, and disulfide between pregnant women with PE and those without PE. Results were pooled using a random-effects model to account for study heterogeneity. The analysis included a total of 631 women diagnosed with PE and 668 healthy pregnant women, encompassing 13 case-control studies and 1 prospective study. Pooled outcomes revealed that women with PE had significantly lower blood levels of native thiol (mean difference [MD] −51.42 umol/L; 95% confidence interval [CI] −79.75 to −23.10 umol/L; *P* < 0.001; *I*^2^ ═ 0%) and total thiol (MD −65.56 umol/L; 95% CI −104.97 to −26.15 umol/L; *P* ═ 0.001; *I*^2^ ═ 0%) compared to the control group. In contrast, no significant difference was observed in blood disulfide levels between the two groups (MD −1.10 umol/L; 95% CI −4.41 to −2.21 umol/L; *P* ═ 0.51; *I*^2^ ═ 0%). Subgroup analyses indicated that the results were consistent across studies matched by gestational age and body mass index, as well as those with varying quality scores (*P* for subgroup differences all > 0.05). In conclusion, women with PE are associated with significantly reduced blood levels of native and total thiols but show no change in blood disulfide levels, suggesting a state of reduced antioxidants in PE.

## Introduction

Preeclampsia (PE) is a significant pregnancy complication characterized by elevated blood pressure and proteinuria, occurring after 20 weeks of gestation [1, 2]. Previous studies have reported varying incidence rates of PE, ranging from 3% to 8% among pregnant women [3]. Clinically, PE is associated with numerous adverse outcomes for both the mother and the fetus [4]. Maternal complications include renal injury, neurological impairment, and eclamptic seizures during pregnancy, while fetal complications encompass preterm birth and low birth weight [4, 5]. Additionally, women with PE are more likely to suffer adverse cardiovascular events later in life [6, 7]. Despite these challenges, the most effective treatment for PE often involves delivering the baby prematurely, leading to iatrogenic preterm birth [8]. Therefore, ongoing efforts are being made to understand the key mechanisms underlying the pathogenesis of PE, aiming to discover the potentially effective preventative and therapeutic strategies [9].

Accumulating evidence suggests that various factors contribute to the pathogenesis of PE, including endothelial dysfunction, chronic inflammation, and oxidative stress, among others [10, 11]. In humans, reactive oxygen species (ROSs) are normally generated during metabolic processes, such as protein synthesis and mitochondrial metabolism. These ROS are neutralized by antioxidants to maintain homeostasis [12, 13]. Thiols are sulfur-containing molecules found within cysteine structures (–S–S) [14]. Total thiols can participate in glutathione metabolism in two ways: either as free radicals or as cysteine groups bound to proteins [14]. Despite composing only 3% of proteins, cysteine groups play a crucial role in various cellular functions, such as signal transduction, molecular stability, metal complex formation, and antioxidant defense [14]. The term “thiol pool” refers to the abundance of cysteine groups in a particular cellular compartment [14]. There is increasing evidence to suggest that disorders related to oxidative stress, including diabetes mellitus, hypertension, inflammatory bowel diseases, and non-small cell lung cancer, may be triggered by disruptions in thiol/disulfide homeostasis [15]. Generally, lower native and total thiol levels in the blood indicate reduced antioxidant capacity, while higher disulfide levels signify increased oxidative stress [16]. Despite these findings, a comprehensive investigation into alterations in thiol–disulfide homeostasis among women with PE remains lacking. To address this gap, the present study conducted a meta-analysis of observational studies to compare blood levels of native thiol, total thiol, and disulfide between women with and without PE.

## Materials and methods

The present study followed the guidelines outlined in the Preferred Reporting Items for Systematic Reviews and Meta-Analyses (PRISMA 2020) [17, 18] and the Cochrane Handbook for Systematic Reviews and Meta-analyses (Handbook for Systematic Reviews and Meta-analysis) [19].

### Literature search

We conducted a comprehensive literature search using Medline, Web of Science, and Embase databases. Our search strategy involved combining keywords related to thiol and disulfide compounds, as well as terms associated with PE, such as PE, eclampsia, pregnancy-induced hypertension, toxemia, edema–proteinuria–hypertension, and edema–proteinuria–hypertension gestosis. Only studies involving human subjects and published in English or Chinese were included. Additionally, we manually screened references of relevant original and review articles to ensure a thorough search. The literature searches were last performed on 22 March 2023.

### Selection of studies

The inclusion criteria for the analysis encompassed the following aspects: inclusion of full-length articles, inclusion of women with PE and healthy pregnant women without PE as cases and controls, respectively, measurement of at least one component of thiol–disulfide homeostasis in both cases and controls, specifically blood levels of native thiol, total thiol, and disulfide and reporting of blood native thiol, total thiol, or disulfide values in means with standard deviations (SDs), or the ability to calculate these values. The diagnosis of PE was determined based on the criteria outlined in the included studies. Preclinical investigations, literature reviews, studies lacking cases of PE, studies lacking normal pregnant control groups, or studies failing to report the blood levels of native thiol, total thiol, or disulfide between cases and controls were excluded. The meta-analysis of studies with overlapping patients included those with the most substantial sample sizes.

### Data collection and study quality assessment

Two authors conducted independent searches, collected data, and evaluated it. Disagreements were resolved through discussions with the corresponding author. The collected data encompassed the following aspects: (1) study details including author, year, and location; (2) participant characteristics, such as case and control numbers, as well as their mean ages; (3) diagnostic criteria for PE in each study; (4) source and characteristics of controls; (5) timing of blood sampling, components of blood thiol–disulfide homeostasis measured, and methods employed; and (6) variables matched or controlled between cases and controls. The study quality was evaluated using the Newcastle--Ottawa Scale (NOS) [20], which assesses three main criteria: the selection of cases and controls, comparability between groups, and exposure measurement. Each study was assigned a score ranging from one to nine, with a higher score indicating a higher quality of the study.

### Ethical statement

In accordance with local/national guidelines, ethical approval was deemed unnecessary for this study. Likewise, written informed consent to participate in the study was not mandated in accordance with the aforementioned guidelines.

### Statistical analysis

The mean difference (MD) and corresponding 95% confidence interval (CI) were used to present the disparity in blood levels of native thiol, total thiol, and disulfide between pregnant women with and without PE [21]. To assess the heterogeneity among studies, the Cochrane’s *Q* test and *I*^2^ statistic were employed as previously described [21, 22]. Heterogeneity was considered present if *I*^2^ > 50%. A random-effects model was utilized to combine the results, accounting for potential heterogeneity between studies [19]. An analysis was conducted to assess the stability of the results by systematically excluding one study at a time. Sensitivity analyses were performed to evaluate the robustness of the findings, specifically focusing on case-control studies, studies diagnosing PE with the American College of Obstetricians and Gynecologists (ACOG) criteria [23], and studies utilizing the automated spectrophotometric method by Erel and Neselioglu [24] to measure blood thiols. Furthermore, subgroup analyses were performed to investigate the impact of study characteristics on the outcomes, including the alignment of gestational age (GA), body mass index (BMI), and quality scores. To assess the presence of publication bias, funnel plots were constructed and visually inspected for symmetry [25]. Additionally, Egger’s regression analysis was employed to test for publication bias [25]. Statistical analysis was conducted using RevMan (Version 5.1; Cochrane Collaboration, Oxford, UK) and Stata. Statistical significance was determined by *P* values less than 0.05.

## Results

### Study retrieval

**Figure 1.**
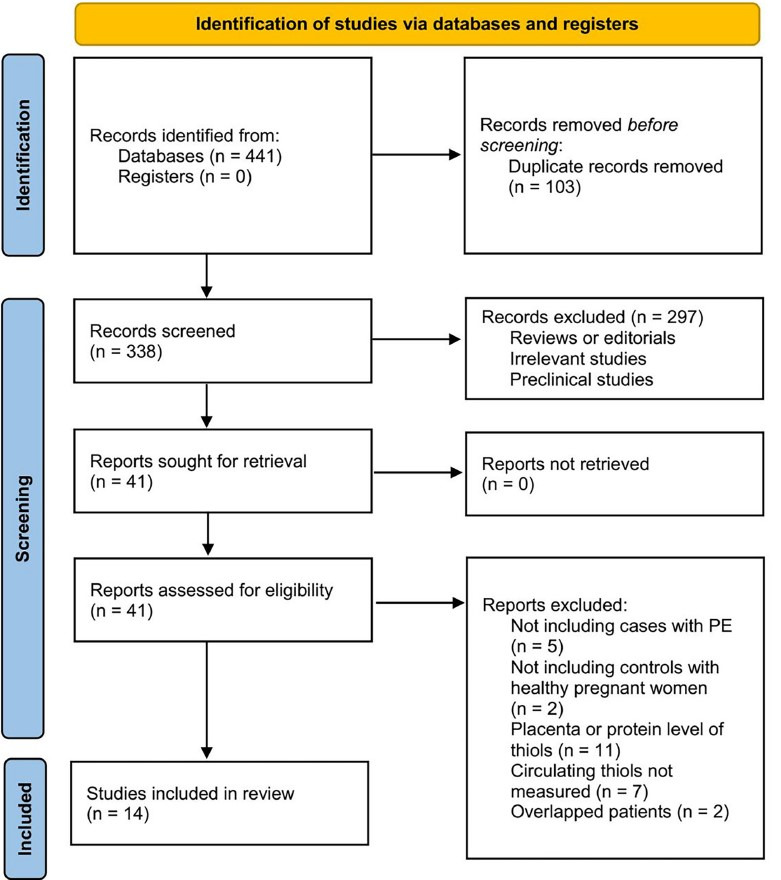
**Diagram illustrating the process of searching databases and identifying studies.** PE: Preeclampsia.

As shown in Figure 1, a comprehensive search of electronic databases yielded a total of 441 articles, which were subsequently reduced to 338 after eliminating duplicates. Out of 338 titles and abstracts screened for inclusion in the meta-analysis, 297 were excluded due to their failure to meet the predetermined criteria. Following an independent evaluation of the full texts by two authors, 27 out of the remaining 41 studies were excluded based on the reasons specified in Figure 1. Consequently, a total of 14 observational studies [26–39] were included in the meta-analysis.

### Study characteristics

Table 1 provides a comprehensive overview of the key attributes of the studies included in the meta-analysis. The analysis encompassed 13 case-control studies [26–37, 39] and one prospective study [38], involving a total of 631 women diagnosed with PE and 668 healthy pregnant women. These studies were conducted in multiple countries—such as the United States, India, Spain, Turkey, Brazil, and The Netherlands, spanning from 1997 to 2022. Participants’ mean ages ranged from 22.3 to 31.3 years. The diagnosis of PE in 12 studies followed the criteria established by the ACOG [26, 27, 29–35, 37–39]. Two studies adhered to the guidelines of the International Society for the Study of Hypertension in Pregnancy (ISSHP) [28, 36]. Blood thiol levels were measured at admission for all but one study. In the prospective study, thiol levels were assessed during the first trimester, prior to the diagnosis of PE [38]. The method of Erel and Neselioglu, employing automated spectrophotometry [24], was used to obtain blood thiol levels in nine of the included studies [29, 31, 32, 34–39]. Variables, such as maternal age, parity, GA, and BMI, were either matched or controlled to a varying degree between pregnant women with and without PE among the included studies. In terms of study quality, most included studies received NOS scores of eight or nine stars, indicating high-quality research (as detailed in Table 2).

**Table 1:** Characteristics of the included studies

**Study**	**Country**	**Study design**	**Maternal age (years)**	**Diagnosis of PE**	**Source of control**	**Number of PE**	**Number of control**	**Timing of sampling**	**Methods for thiol measuring**	**Differences in thiol reported**	**Variables matched or adjusted**
Hubel 1997	USA	Case-control	Mean: 26.4	ACOG	Healthy pregnant women	14	17	At admission	EPR spectroscopy techniques	Total thiol	Age, BMI, and prepregnant BP
Kharb 2000	India	Case-control	Range: 19--36, Mean: 22.3	ACOG	Normotensive pregnant women	30	30	At admission	Colorimetry	Native thiol and total thiol	Age, HGB, and GA
Llurba 2004	Spain	Case-control	Mean: 30.5	ISSHP	Healthy pregnant women	53	30	At admission	Colorimetry	Native thiol	Age, parity, BMI, and GA
Ozler 2015	Turkey	Case-control	Mean: 30.4	ACOG	Healthy pregnant women	43	43	At admission	Automated spectrophotometric method	Native thiol, total thiol, and disulfide	Age, parity, and BMI
de Lucca 2016	Brazil	Case-control	Mean: 27.2	ACOG	Healthy pregnant women	55	30	At admission	Colorimetry	Native thiol	Age, GA, and BMI
Korkmaz 2016	Turkey	Case-control	Mean: 27.8	ACOG	Pregnant women without obstetric complications	71	37	At admission	Automated spectrophotometric method	Native thiol, total thiol, and disulfide	Age and parity
Yuvacı 2016	Turkey	Case-control	Range: 18--40, Mean: 28.8	ACOG	Healthy pregnant women	62	37	At admission	Automated spectrophotometric method	Native thiol, total thiol, and disulfide	Age, gravidity, and parity
Bos 2019	The Netherlands	Case-control	Mean: 31.3	ACOG	Women with uncomplicated pregnancy	24	23	At admission	Ion-exchange chromatography	Native thiol	Age and BMI
Cakar 2019	Turkey	Case-control	Mean: 29.2	ACOG	Healthy pregnant women	51	61	At admission	Automated spectrophotometric method	Native thiol, total thiol, and disulfide	Age, parity, BMI, and GA
Kaya 2020	Turkey	Case-control	Mean: 28.1	ACOG	Pregnant women without obstetric complications	50	30	At admission	Automated spectrophotometric method	Native thiol, total thiol, and disulfide	Age, BMI, and platelet count
Onat 2020	Turkey	Case-control	Mean 28.7	ISSHP	Normotensive pregnant women	47	57	At admission	Automated spectrophotometric method	Native thiol, total thiol, and disulfide	Age, gravidity, parity, BMI, and GA
Tasan 2021	Turkey	Prospective	Mean: 29.4	ACOG	Healthy pregnant women	38	177	Before diagnosis (first trimester)	Automated spectrophotometric method	Native thiol, total thiol, and disulfide	Age and GA
Ovayolu 2021	Turkey	Case-control	Mean: 26	ACOG	Healthy pregnant women	50	51	At admission	Automated spectrophotometric method	Native thiol, total thiol, and disulfide	GA and gravidity
Gul 2022	Turkey	Case-control	Mean: 28.1	ACOG	Normotensive pregnant women	43	45	At admission (third trimester)	Automated spectrophotometric method	Native thiol, total thiol, and disulfide	Age, GA, and BMI

PE: Preeclampsia; EPR: Electron paramagnetic resonance; ACOG: The American College of Obstetricians and Gynecologists; ISSHP: The International Society for the Study of Hypertension in Pregnancy; BMI: Body mass index; GA: Gestational age; HGB: Hemoglobin; BP: Blood pressure.

**Table 2:** Study quality evaluation via the Newcastle--Ottawa Scale

	**Adequate definition of the cases**	**Representativeness of the cases**	**Selection of controls**	**Definition of controls**	**Controlled for GA**	**Controlled for other factors**	**Ascertainment of the exposure**	**Same method of ascertainment of exposure for cases and controls**	**Non-response rate**	**Overall**
Hubel 1997	1	1	1	1	0	1	1	1	1	8
Kharb 2000	1	1	1	1	1	1	1	1	1	9
Llurba 2004	1	1	1	1	1	1	1	1	1	9
Ozler 2015	1	1	1	1	0	1	1	1	1	8
de Lucca 2016	1	1	1	1	1	1	1	1	1	9
Korkmaz 2016	1	1	1	1	0	1	1	1	1	8
Yuvacı 2016	1	1	1	1	0	1	1	1	1	8
Bos 2019	1	1	1	1	0	1	1	1	1	8
Cakar 2019	1	1	1	1	1	1	1	1	1	9
Kaya 2020	1	1	1	1	0	1	1	1	1	8
Onat 2020	1	1	1	1	1	1	1	1	1	9
Tasan 2021	1	1	1	1	1	1	1	1	1	9
Ovayolu 2021	1	1	1	0	1	1	1	1	1	8
Gul 2022	1	1	1	1	1	1	1	1	1	9

GA: Gestational age.

### Difference of native thiol between women with and without preeclampsia

**Figure 2.**
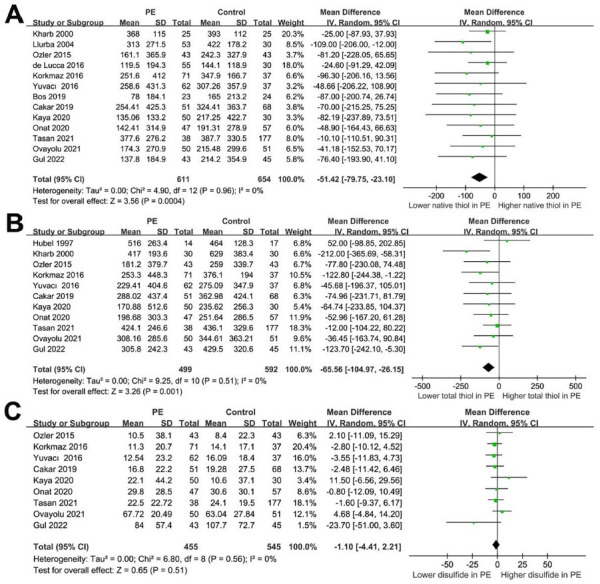
**Forest plots representing the changes in thiol–disulfide homeostasis in women with PE on the basis of a meta-analysis.** (A) Blood native thiol levels compared in women with and without PE; (B) Blood total thiol levels compared in women with and without PE; (C) Blood native disulfide levels compared in women with and without PE. PE: Preeclampsia; CI: Confidence interval; SD: Standard deviation; chi^2^: Chi-squared; tau^2^: Tau-squared.

Pooled results from 13 studies [27–39] revealed that, compared to the control group, women with PE had significantly lower levels of native thiol in the blood (MD −51.42 umol/L, 95% CI −79.75 to −23.10, *P* < 0.001) (Figure 2A). There was no significant heterogeneity observed (Cochrane’s *Q* test *P* ═ 0.96, *I*^2^ ═ 0%). A consistent result was obtained by excluding one study at a time in the influencing analysis (MD −46.05 to −58.14 umol/L, all *P* < 0.05). Further sensitivity analyses that were limited to case-control studies (MD −54.99 umol/L, *P* < 0.001), studies diagnosing PE according to ACOG criteria (MD −45.85 umol/L, *P* ═ 0.003), and studies using the automated spectrophotometric method for measuring thiols (MD −57.97 umol/L, *P* ═ 0.006), also showed consistent results (Table 3).

**Table 3:** Sensitivity and subgroup analyses

	**Native thiol (umol/L)**				**Total thiol (umol/L)**				**Disulfide (umol/L)**			
**Characteristics**	**Number of studies**	**MD (95% CI)**	***P*1**	***P*2**	**Number of studies**	**MD (95% CI)**	***P*1**	***P*2**	**Number of studies**	**MD (95% CI)**	***P*1**	***P*2**
*Study design*												
Only case-control studies	12	−54.99 (−84.52, −25.47)	<0.001	NA	10	−77.52 (−121.11, −33.94)	<0.001	NA	8	−0.99 (−4.65, 2.66)	0.59	NA
*Diagnosis of PE*												
Only with ACOG criteria	11	−45.85 (−76.49, −15.21)	0.003	NA	10	−67.47 (−109.99, −24.96)	0.002	NA	8	−1.13 (−4.59, 2.33)	0.52	NA
*Thiol measuring methods*												
Only automated spectrophotometric method	9	−57.97 (−99.30, −16.64)	0.006	NA	9	−63.71 (−106.05, −21.36)	0.003	NA	9	−1.10 (−4.41, 2.21)	0.51	NA
*GA matched*												
Yes	8	−42.02 (−74.33, −9.72)	0.01		6	−72.33 (−126.80, −17.87)	0.009		5	−0.91 (−5.53, 3.71)	0.70	
No	5	−82.69 (−141.61, −23.77)	0.006	0.24	5	−58.42 (−123.83, 6.99)	0.08	0.75	4	−1.35 (−6.22, 3.52)	0.59	0.90
*BMI matched*												
Yes	8	−62.99 (−101.03, −24.94)	0.001		6	−62.01 (−118.74, −5.28)	0.03		5	−0.63 (−7.16, 5.90)	0.85	
No	5	−37.04 (−79.47, 5.39)	0.09	0.37	5	−75.01 (−143.07, −6.95)	0.03	0.77	4	−1.30 (−5.35, 2.76)	0.53	0.87
*Quality scores*												
8	6	−73.61 (−125.69, −21.53)	0.006		6	−53.83 (−112.01, 4.35)	0.07		5	−0.10 (−4.44, 4.24)	0.96	
9	7	−42.10 (−75.85, −8.34)	0.01	0.32	5	−81.62 (−146.66, −16.58)	0.01	0.53	4	−2.50 (−7.61, 2.61)	0.34	0.48

*P*1: *P* for effect of subgroup; *P*2: *P* for subgroup interaction; MD: Mean difference; CI: Confidence interval; ACOG: The American College of Obstetricians and Gynecologists; ISSHP: The International Society for the Study of Hypertension in Pregnancy; BMI: Body mass index; GA: Gestational age; PE: Preeclampsia.

Subgroup analyses indicated that the differences in native thiol levels between pregnant women with and without PE were not significantly influenced by matching GA, BMI, or varying study quality scores (all *P* for subgroup differences > 0.05) (Table 3).

### Difference of total thiol between women with and without preeclampsia

Meta-analysis including 11 studies [26, 27, 29, 31, 32, 34–39] showed that women with PE had a significantly lower blood level of total thiol (MD −65.56 umol/L, 95% CI −104.97 to −26.15, *P* ═ 0.001, *I*^2^ ═ 0%) (Figure 2B). Results were similar in influencing analysis via omitting one study at a time (MD −55.26 to −74.17 umol/L, all *P* < 0.05). Sensitivity analyses limited to case-control studies, studies with ACOG-defined PE, and studies with the automated spectrophotometric method also showed consistent results (MD −77.52 to −64.74, and −63.71 umol/L, respectively, all *P* < 0.05) (Table 3). Besides, subgroup analyses showed that study characteristics, such as matching GA or BMI between groups, or the difference in quality scores, were not likely to affect the results (all *P* for subgroup differences > 0.05) (Table 3).

### Difference of disulfide between women with and without preeclampsia

Pooling the results of nine studies [29, 31, 32, 34–39] indicated that the blood level of disulfide was not significantly different between cases and controls (MD −1.10 umol/L, 95% CI −4.41 to −2.21, *P* ═ 0.51, *I*^2^ ═ 0%) (Figure 2C). Influencing analysis by omitting one study at a time showed consistent results (MD −0.67 to −1.54 umol/L, all *P* > 0.05). Sensitivity analyses limited to case-control studies, studies with ACOG-defined PE, and studies with the automated spectrophotometric method also showed similar results (all *P* > 0.05) (Table 3). Additionally, subgroup analyses showed consistent results in studies with the match of GA and BMI, and in studies with various quality scores (all *P* for subgroup differences > 0.05) (Table 3).

### Publication bias

**Figure 3.**
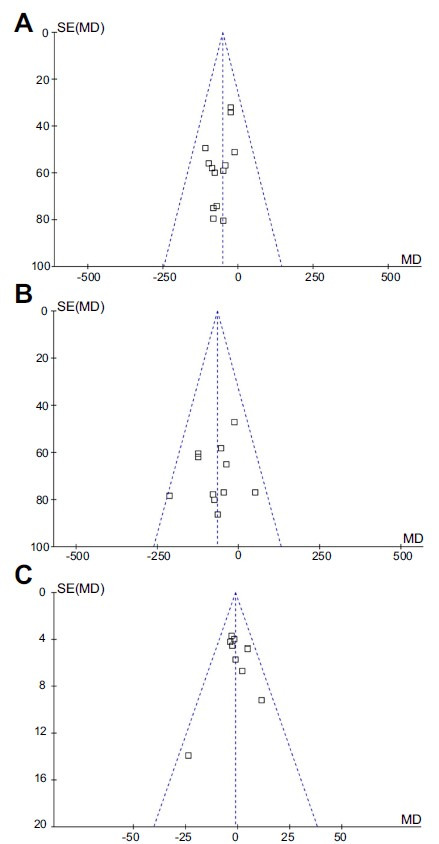
**An analysis of the publication bias of the meta-analyses based on funnel plots.** (A) Blood native thiol levels compared in women with and without PE; (B) Blood total thiol levels compared in women with and without PE; (C) Blood disulfide levels compared in women with and without PE. MD: Mean difference; PE: Preeclampsia.

The funnel plots depicting the meta-analyses examining the blood levels of native thiol, total thiol, and disulfide are presented in Figure 3A–3C. The symmetrical nature of these plots suggests a minimal likelihood of publication bias. Furthermore, the application of Egger’s regression tests yielded non-significant results for publication bias (*P* ═ 0.57, 0.82, and 0.21, respectively).

## Discussion

Our meta-analysis consolidated the results of 14 observational studies, revealing that women with PE had significantly lower native and total thiol levels in their blood compared to women with normal pregnancies. Interestingly, disulfide levels did not differ significantly between the two groups. To ensure the robustness of our findings, we conducted a leave-one-out analysis and multiple sensitivity analyses, including analyses restricted to case-control studies, studies adhering to ACOG criteria for PE diagnosis, and studies using automated spectrophotometric methods for thiol measurement. Subgroup analyses indicated that the difference of native thiol, total thiol, and disulfide between pregnant women with and without PE was not significantly affected by match of GA, BMI, or different quality scores. Taken together, our meta-analysis demonstrates that women with PE are associated with significantly reduced blood native and total thiols, but unchanged blood disulfide, suggesting a state of reduced antioxidants in women with PE.

Until now, no meta-analysis has comprehensively assessed changes in thiol–disulfide homeostasis among women with PE. Our study has several methodological strengths that warrant attention before interpreting the results. First, we conducted a comprehensive search across three electronic databases, yielding 14 up-to-date observational studies pertinent to our research objectives. Second, we separately analyzed three key components of blood thiol–disulfide homeostasis, including native thiol, total thiol, and blood disulfide—which allows for a nuanced understanding of changes in the antioxidant–ROS balance in women with PE. Third, we took steps to minimize confounding by matching or controlling for variables, such as maternal age, GA, parity, and BMI in the included studies. Lastly, our findings were consistent across various influencing analyses, sensitivity analyses, and multiple predefined subgroup analyses, affirming the robustness and stability of our conclusions. In summary, our meta-analysis showed that women with PE had significantly reduced levels of native and total thiols in their blood compared to women without PE, while disulfide levels remain unchanged. These findings point toward an imbalanced thiol–disulfide homeostasis, favoring a state of oxidative stress in women with PE.

Although oxidative stress injury has been proposed as an underlying mechanism of PE, a reliable and comprehensive assessment for the antioxidant/ROS balance in women with PE has rarely been performed [40]. With the recent understanding regarding the changes of thiol–disulfide homeostasis underlying the pathogenesis of multiple diseases, particularly with the convenient assessment methods proposed by Erel and Neselioglu with the automated spectrophotometrics [24], an overview of the changes of thiol–disulfide homeostasis in women with PE become feasible. Results of the meta-analysis showed that women with PE had significantly lower blood levels of native and total thiols, indicating inadequate antioxidants in women with PE. It may be because of the fact that oxidative pathways have been stimulated as the disease progresses, which means that antioxidant effectors, such as thiols, will be depleted to buffer the oxidative stress [41]. In addition, proteinuria in women with PE leads to albumin loss, and as albumin is the main carrier protein for thiol groups in the plasma, total and native thiol concentrations may reduce subsequently [42]. These findings may also explain the findings of some previous clinical studies that supplementation with some antioxidants may be effective in reducing the incidence of PE in high-risk women [43, 44]. In addition, we found that the blood level of disulfide was not significantly different between women with and without PE. Generally, serum disulfide levels result from disulfide bonds occurring within cysteine groups of serum proteins (primarily albumin). When ROS are increased, reduced thiol groups become oxidized and form disulfide bonds [45]. Accordingly, increased serum disulfide levels may be expected in women with severe PE, in whom the consumption of antioxidant could not neutralize the enhanced ROS generation. Although the exact mechanisms underlying the changes of thiol–disulfide homeostasis in women with PE should be determined in future studies, assessment of thiol–disulfide homeostasis in individual high-risk women may be important for estimating the risk of PE and formulating preventative and therapeutic strategies. Studies are also warranted to explore the clinical implications for evaluating the changes of thiol–disulfide homeostasis in women with PE.

There are several limitations inherent in our study. First, the number of included studies and patients is limited, necessitating the validation of our findings through large-scale research. Second, the majority of the included studies were case-control studies, highlighting the need for extensive prospective studies to ascertain the independent association between changes in thiol–disulfide homeostasis and PE. Additionally, as a meta-analysis of observational studies, our study was unable to establish a causal relationship between the incidence of PE and alterations in thiol–disulfide homeostasis. Last, despite efforts to match or control for certain confounding factors in women with and without PE, it is important to acknowledge the possibility of remaining residual factors that could impact the relationship between blood thiols and PE, such as variations in nutritional status and dietary patterns.

## Conclusion

In summary, the presence of PE in women is linked to a notable decrease in blood native and total thiols, while blood disulfide remains unaltered. This indicates a diminished level of antioxidants in individuals with PE. To fully comprehend the clinical significance of evaluating thiol–disulfide homeostasis in women with PE, additional research endeavors are imperative.
